# Comparison of the External Load in Training Sessions and Official Matches in Female Football: A Case Report

**DOI:** 10.3390/ijerph192315820

**Published:** 2022-11-28

**Authors:** Aratz Olaizola, Ibai Errekagorri, Karmele Lopez-de-Ipina, Pilar María Calvo, Julen Castellano

**Affiliations:** 1 Department of Physical Education and Sport, Faculty of Education and Sport, University of the Basque Country (UPV/EHU), 01007 Vitoria-Gasteiz, Spain; 2 Society, Sports and Physical Exercise Research Group (GIKAFIT), Department of Physical Education and Sport, Faculty of Education and Sport, University of the Basque Country (UPV/EHU), 01007 Vitoria-Gasteiz, Spain; 3 Department of Psychiatry, Cambridge Neuroscience, University of Cambridge, Cambridge 01223, UK; 4 Department of Computers’ Arquitecture and Technology, University of the Basque Country (UPV/EHU), Paseo M. Lardizabal, 1, 20018 San Sebastian, Spain

**Keywords:** team sport, women, external load, periodization, electronic performance, tracking systems

## Abstract

The objective of this study was to compare the external load of training sessions using as a reference an official competition match in women’s football in order to find if the training sessions replicate the competition demands. Twenty-two semi-professional football players were analyzed during 17 weeks in the first phase of the competitive period of the 2020–2021 season of Spanish women’s football. In addition to the competition (Official Matches, OM), four types of sessions were distinguished: strength or intensity (INT), endurance or extensity (EXT), velocity (VEL), and activation or pre-competitive (PREOM). The external load variables recorded were total distance (TD), high-speed running (HSR), sprint (Sprint), accelerations (ACC2), decelerations (DEC2), player load (PL), distance covered per minute (TDmin), high metabolic load distance (HMLD), and total impacts. The main results were that the external load demanded was different according to the type of session, being, in all cases, much lower than OM. The variables referring to the neuromuscular demands (ACC2 and DEC2) were higher in the INT sessions, the TD variable in the EXT sessions and the velocity variables (HSR and Sprint) in the VEL sessions. We can conclude that there was an alternating horizontal distribution of training loads within the competitive micro-cycle in women’s football, although the order was not the usual one for tactical periodization.

## 1. Introduction

In recent decades, there has been exponential global development of women’s football, both in its practice and in the entities and institutions that promote and manage it [1,2]. This has been accompanied by a greater professionalization in elite game standards, in addition to an increase in audiences, leading to the creation of professional leagues and clubs, generating greater professionalization [3]. The scientific interest in women’s football has not been left out of this reality, which is why competition analysis is now a focus with the aim of deepening its knowledge [4,5,6,7]. 

It is essential to know the demands of women’s competition [8] in order to have reference values to guide training content. In a complementary way, the evaluation of the training process is essential to verify the effectiveness of the intervention and to search for the best strategy to stimulate the athlete, that is, to distribute training and recovery [9] in order to optimize physical condition [10]. It is also important to pay attention to load management in order to reduce the probability that players could suffer over-training or even injury [11,12,13].

In men’s football, the two usual planning strategies are the structured micro-cycle together with tactical periodization [10,14]. Elite football teams use the latter because the proposed content prioritizes technical/tactical objectives over conditional and psychological capabilities simultaneously [15]. Previous studies [4,14,16,17,18,19] agree that the planning strategy of a competitive micro-cycle with a single competitive game is three main acquisition sessions during the week in order to prepare and sustain physical abilities, such as strength, endurance, and velocity. This first part of acquisition is followed by a load reduction phase at the end of the week (tapering phase) to ensure greater freshness and, therefore, a greater willingness to compete [18,20]. Both phases result in a horizontal alternation in the distribution of conditional demands during the micro-cycle [16,17].

In order to improve the knowledge of types of load management strategies in women’s football in a novel way, the objective of the present study is to describe the external load in the different types of training and competitive matches in women’s football during a competitive micro-cycle. The starting hypothesis is that there is a horizontal alternation in the conditional demand between the training sessions in the competitive micro-cycle, but none of the sessions replicates the competition demand. In this sense, the results of this study will allow increasing the information on the type of periodization that is proposed in an elite women’s football team, which could help to facilitate load management in women’s football, which is still little known and under research.

## 2. Materials and Methods

### 2.1. Participants

A total of 22 semi-professional female football players (age: 24.6 ± 4.0 years; height: 163.9 ± 5.0 cm; weight: 58.5 ± 4.2 kg; skinfolds (i.e., the sum of 6 skinfolds: triceps, subscapular, supraspinal, abdominal, front thigh, and medial calf): 65.4 ± 17.9 mm) took part in the study. The data recording was carried out during 17 weeks of the competitive period of the Second Women’s Football Division (Reto Iberdrola) during the 2020–2021 season. Usually, the team completed four days of training and one day of competition per week.

### 2.2. Procedures

In order to obtain position data, the players were monitored with WIMU PRO devices (RealTrack Systems, Almeria, Spain) using the global positioning system (GPS). The GPS device used in this study can operate at 10 Hz, and it is compatible with the Galileo and American satellite constellation, which seems to provide more precision [21]. For the analysis, data were collected on outdoor football fields without any possibility of infrastructure interfering with the data collection. During the sessions, a mean of 12 satellites were connected with each device. The value of DDOP was 0.95. This equipment and its measurements are valid and reliable using the GNSS for time-motion analysis in football (distance covered variable: accuracy = 0.69–6.05%, test–retest reliability = 1.47, inter-unit reliability = 0.25; mean velocity variable: accuracy = 0.18, intra-class correlation = 0.95, inter-unit reliability = 0.03) [22], and has been awarded with the FIFA Quality Performance certificate. Each WIMU PRO device was placed in a vertical position between the players’ shoulder blades, in a pocket of a specific chest vest (dimensions of the devices = 81 × 45 × 16 mm). The GPS devices were activated 15 min before the start of each session or match in accordance with the manufacturer’s instructions. All the players were familiar with the use of GPS. Only the players that completed official matches or training sessions were included in the analysis. To avoid possible differences between devices, during the entire registration period, each player wore the same device [23,24]. The records were downloaded using the SPRO software (RealTrack Systems, Almeria, Spain) after the end of each session. Once the data were filtered through the software, they were imported into a Microsoft Excel spreadsheet (Microsoft Corporation, Washington, DC, USA) to configure a matrix. 

### 2.3. Physical Variables

The duration of the session was recorded considering only the effective time, that is, the time in which the players were active, excluding times of inactivity (e.g., stoppages between tasks). The external load variables were: total distance (TD, in m); high speed running (HSR, in m), established as 60% of the maximum individual velocity of the participants [25]; sprint (Sprint, in m), defined as the distance accumulated above 85% of the maximum individual velocity [26]; accelerations over 2 m/s^2^ (ACC2, in n); decelerations of less than −2 m/s^2^ (DEC2, in n); player load (PL, in au), distance traveled per minute (TDmin, in m/min^1^); high metabolic load distance (HMLD, in m), defined as the distance covered by a player when their metabolic power is above 25.5 W/kg^1^, ratios per kilogram [14]; and, total impacts (Total Impacts, in n) using an Earth sensor to calculate the three axes value of the module. As for the choice of the maximum individual velocity, the highest value recorded during the 17-week period of the study was chosen, considering both the training sessions and the competition. 

### 2.4. Type of Training Sessions and Official Matches

Apart from the official matches (OM, *n* = 12), the types of sessions were differentiated based on the priority conditional objective that was developed: strength or intensity session (INT, *n* = 10); endurance or extensity session (EXT, *n* = 15); velocity session (VEL, *n* = 7); and activation session (PREOM, *n*= 14). In total, 805 recordings were collected from official matches (*n* = 49) and 756 training sessions, distributed as follows: VEL = 114, INT = 173, EXT = 239, and PREOM = 230. The number of records per player was 36.8 ± 10.6.

The order of the sessions in the training micro-cycle was conditioned to the number of days after (OM+) and before (OM−) of the OM, following the proposal of previous studies [10,14].

The VEL session was usually the first acquisition session, located 2 days after the OM (OM + 2) and 5 or 6 days before the next official match (OM − 6 or OM − 5), characterized by velocity work, locomotive-oriented and based on tasks of intermittent nature or in waves (with breaks of 1 or 2 min between drills). The tasks were mostly carried out with goalposts and goalkeepers and were made up of a large relative space per player (e.g., >250 m^2^). 

The INT session was the second acquisition session of the week, usually located 3 or 4 days after the OM (OM + 3 or OM + 4) and 3 or 4 days before the next competition (OM − 3 or OM − 4), characterized by neuromuscular and mechanically-oriented work, based on reduced and positional games, with a relative space per player of less than 100 m^2^ and with a number of participants ranging from one to six per team.

The EXT session was usually the third acquisition session of the micro-cycle, located 4 or 5 days after the OM (OM + 4 or OM + 5) and 2 or 3 days before the next competition (OM − 2 or OM − 3), characterized by cardiovascular work, based on large-format tasks, that is, with a moderate to high number of participants per team (>6), in a relative space equal to or greater than 250 m^2^ per player, and with goalposts and goalkeepers.

Pre-match day (PREOM) was the fine-tuning session. It was always conducted the day before match day (OM − 1). The contents that were developed in it were activation tasks based on activities with a large number of participants (>6) in a medium relative space (equal to or less than 250 m^2^ per player) or small (equal to or less than 100 m^2^ per player) with a polarized orientation and a tactical and strategic approach. 

The last day of the week was match day (OM). To calculate the load on the day of the competition, the previous warm-up carried out by the players was taken into account in addition to the two parts of the match, which had the following approximate load: duration was 20.1 ± 2.1 min, and the physical demands were as follows: TD: 1280 ± 266 m, HSR: 64.3 ± 37.7 m, Sprint: 5 ± 10.7 m, ACC2: 27.9 ± 9.6 n, DEC2: 28.4 ± 9.6 n, PL 20.6 ± 4.3 au and TDmin 64 ± 12.3 (m/min^1^).

### 2.5. Statistical Analysis

Descriptive statistics data from variables were presented using mean and standard deviation. Tests for normality (Shapiro–Wilk) and equality of variances (Levene) were applied. The null hypothesis was accepted because the distribution of the data met the normality criterion. Furthermore, the variances were homogeneous. Therefore, a one-way ANOVA analysis of variance for independent samples was used to test for differences in the variables between the different sessions (INT, EXT, VEL, PREOM, and OM). Significant results were then analyzed using post hoc Tukey’s test. Effect size (ES) was also calculated to determine meaningful differences with magnitudes classified as [27] trivial (<0.2), small (>0.2–0.6), moderate (>0.6–1.2), large (>1.2–2.0), and very large (>2.0–4.0). The level of significance was set at *p* < 0.05. The statistical analysis was conducted using the software JASP 0.14.1 (University of Amsterdam, Amsterdam, The Netherlands) and a customized Microsoft Excel spreadsheet (Microsoft Corporation, Washington, DC, USA) for Windows.

## 3. Results

**Figure 1:**
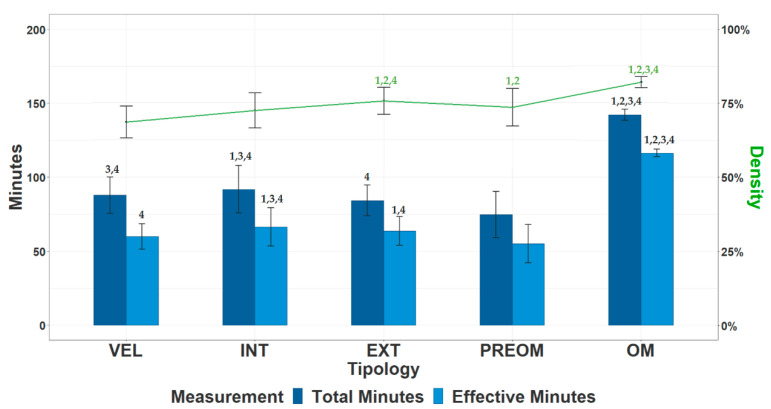
Total duration (min, in dark blue), effective duration (min, in light blue), and density (effective/total, %, in green) of the sessions. Note: VEL is velocity day, INT is intensity or strength day, EXT is extensity or endurance day, PREOM is previous day to match day, OM is official match day. Significant differences (*p* < 0.05) are represented: 1 is higher than VEL, 2 is higher than INT, 3 is higher than EXT, and 4 is higher than PREOM.

Figure 1 shows the total time (min), effective time (min), and density (%) of the training and competition sessions. There were significant differences (*p* < 0.05) between training sessions for all variables. The PREOM session had the lowest (*p* < 0.05) volume (total and effective time) but with higher density than the VEL and INT sessions. The INT session had the highest volume (total and effective time) but with a lower density than the EXT and PREOM sessions and higher than VEL.

As shown in Table 1, all external load variables were higher in OM compared to the rest of the training sessions, with a difference ranging from moderate to extremely large (ES = 0.7–6.6). On the contrary, the session with the lowest load in all the variables was the PREOM. The variables ACC2 and DEC2 oriented towards neuromuscular work were higher in INT, the variable TD oriented towards cardiovascular work was higher in EXT, and variables of velocity (HSR and Sprint) oriented towards locomotor work were higher in VEL. The values of the intensity variable (TDmin) were highest in OM with respect to all training sessions.

**Figure 2:**
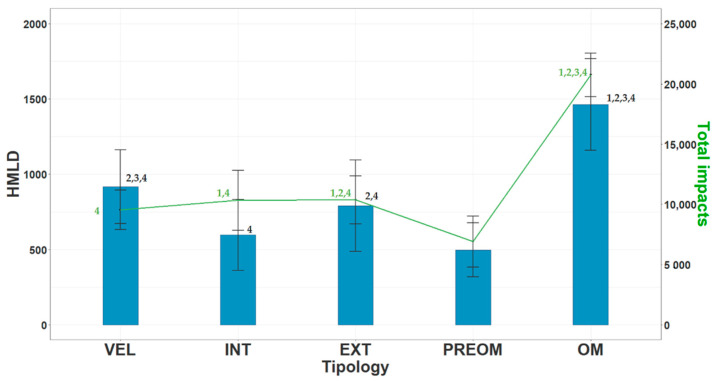
Means and deviations of the high metabolic load distance (HMLD, m, in blue) and total impacts (Total impacts, n, in green) of the players on the different training days. Note: VEL is velocity day, INT is intensity or strength day, EXT is extensity or endurance day, PREOM is previous day to match day, OM is official match day. Significant differences (*p* < 0.05) are represented: 1 is higher than VEL, 2 is higher than INT, 3 is higher than EXT, and 4 is higher than PREOM.

Figure 2 shows the mean and standard deviations of two external load variables: High Metabolic Load Distance (HMLD) and Total Impacts. Significant differences were found for both variables in the different training and competition sessions. In line with the rest of the variables, the highest values were obtained in OM and the lowest in PREOM. The VEL session obtained the highest HMLD values compared to the other training sessions. Likewise, the EXT session accumulated the highest values in Total Impacts compared to the other training sessions.

Table 2 shows the effect size (ES) of the external load variables obtained from the comparison between training sessions and official matches. As can be seen, the magnitude of the differences ranged from a very large decrease (ES = −2.17) to an extremely large increase (ES = 7.4).

## 4. Discussion

The aim of this study is to describe the distribution of external load in different training sessions and competitions in competitive micro-cycles in women’s football. According to the current state of the art, this is the first study carried out on the description of tactical periodization in women’s football. The results confirm the starting hypotheses because despite describing a distribution of the conditional demands (for example, strength, resistance, and speed) based on the horizontal alternation during the competitive micro-cycle sessions, none of them replicated the demands of the competition.

In line with the results obtained in previous studies carried out in men’s football [14,28], the total and effective duration was significantly shorter in the training sessions compared to those obtained in official matches (Figure 1). Among the three acquisition sessions (VEL, INT, and EXT), the VEL session was the one with the lowest density, probably due to the intermittent or dividing nature of the tasks of this session. It should be highlighted as well that the lowest values in all the variables are obtained in the PREOM session, confirming the existence of tapering in the training load in the days prior to competition in order to favor the recovery of the players and, consequently, to guarantee greater freshness and willingness to compete [10,16,17,19]. The results of this study concur with these previous contributions in men’s football, where the weekly periodization was described, accumulating the highest loads in the middle of the week, that is, 2 or 3 days before the match (OM − 2 or OM − 3), coinciding with the EXT day, and lower loads at the end of the week, the 2 days before the competition.

On the other hand, unlike previous studies [10,18], the location of the VEL session within the weekly distribution was novel. This was probably motivated by the distribution of the training sessions that the team arranged, trying to distance the VEL session from the EXT session, thereby stimulating the distance covered at high-speed ranges (e.g., HSR and Sprint) and that of the following match. The reason could lie in the need to emphasize this conditional capacity given its low stimulation both in the competition itself (e.g., players accumulated little distance in high-speed ranges) and throughout the competitive micro-cycle. The VEL session was located on the second day after the match played in the previous micro-cycle (OM + 2), that is, 5 or 6 days prior to the following match (OM − 5 or OM − 6). Significantly more HSR and Sprint were accumulated in these sessions, with a volume load (TD) similar to the EXT session. 

Regarding the INT session, which was usually carried out on the central days of the week (OM − 5 or OM − 4) and prior to the EXT session, a predominance of the force variables (accelerations and decelerations) was described compared to the other sessions, as described in men’s football in previous works [10,29]. However, although Stevens et al. (2017) described that medium (1.5–3 m/s^2^ and −1.5–3 m/s^2^) and high (>3 m/s^2^ and <−3 m/s^2^) accelerations and decelerations during training were similar to the competition values in this type of session, in this research work, the competition values were significantly higher than the rest of the types of training sessions. 

With regard to the EXT session, which was usually carried out in the middle of the week (OM − 4 or OM − 3), high values were described in TD and PL compared to the other sessions, showing a similar trend as in previous studies [18,30]. In elite male football, these studies described greater distances covered in OM − 3 sessions compared to OM − 4 sessions, which could be related to the EXT and INT sessions described in this study.

Despite the global coincidence of this approach, not all the works [17,31,32,33] concurred in the same weekly profile of the loads. While some showed a downward progression or from more to less [31], others described two peaks, Monday and Thursday [33], with a remarkable peak in the main session of the week [32] or with high values in the middle of the week without showing great differences between sessions [17]. Likewise, the present work shows three load sessions (VEL, INT, AND EXT), of which two obtain higher values (VEL and EXT), located at the beginning and middle of the week, as represented in the study by [33]. 

This study describes a variant of tactical periodization, which, while respecting horizontal alternation, proposes a novel distribution in the orientation of content throughout the competitive micro-cycle. The possible practical application of this study is that the content of the training sessions with different conditional orientations could present a different ordering than the one proposed by the tactical periodization. In this way, instead of respecting the usual order of the sessions proposed by the tactical periodization (e.g., INT+EXT+VEL), it could be altered by proposing VEL+INT+EXT when the contextual needs and/or the characteristics of the type of population to which it is directed requires adapting content to optimize their preparation process.

It has been verified that none of the training sessions obtained higher values than those of the competition in all the variables (e.g., oriented towards neuromuscular, cardiovascular, and locomotor works). Therefore, coaches should be careful in the return-to-play process and should prepare players adequately because there could be an excessive gap between the demands of training sessions and those of competition, putting players at risk [11,12,13].

This work has some context limitations. In the first place, since just one team was analyzed, the periodization results should not be interpreted as something generalized in women’s football; more case studies would be required to approach an extrapolation. In addition, the inclusion of internal load variables [34] could have allowed knowing in greater detail the internal response generated in the athlete [35]. Likewise, it could be interesting to carry out a battery of physical tests or to pass wellness and readiness questionnaires to assess the physical condition of the players or their willingness to compete [10,26]. However, it is worth mentioning that the team achieved promotion to the highest category of women’s football at the national level; therefore, it could support the idea that the proposed periodization strategy not only had no negative effects on the performance of the players but also had possibly a great impact on the competitive performance. 

## 5. Conclusions

In conclusion, this research work provides a description of the profile of training loads throughout a competitive week in women’s football. A horizontal alternation in the stimulation of physical capacities in female football was described, although the order of the training contents varied with respect to the original proposal of tactical periodization. This study could open the possibility of proposing a variant that can better adapt to a particular reality conditioned by the schedules and pitches established by the sports club of the players. In this sense, in ongoing works, a larger sample and other contexts will be included.

## Figures and Tables

**Table 1:** Mean and standard deviation (SD) of the external load variables in different training and competition sessions.

Type of Sessions										
	VEL		INT		EXT		PREOM		OM	
Variables	Mean	(SD)	Mean	(SD)	Mean	(SD)	Mean	(SD)	Mean	(SD)
TD	5058.3	(712.3) ^2,3^	4840.0	(1237.8) ^4^	5418.5	(957.6) ^1,2,4^	3546.8	(1007.8)	10,576.33	(602.77) ^1,2,3,4^
(m)										
HSR	614.5	(318.1) ^2,3,4^	184.0	(187.1)	424.3	(247.7) ^2,4^	245.3	(126.0) ^2^	906.7	(208.0) ^1,2,3,4^
(m)										
Sprint	35.1	(49.6) ^2,3,4^	1.5	(5.5)	28.2	(42.4) ^2,4^	7.8	(15.2) ^2^	69.2	(51.2) ^1,2,3,4^
(m)										
ACC2	115.3	(25.2) ^3,4^	145.0	(40.7) ^1,3,4^	99.7	(24.7) ^4^	71.1	(24.2)	181.8	(35.1) ^1,2,3,4^
(n)										
DEC2	118.5	(29.2) ^3,4^	138.1	(39.0) ^1,3,4^	99.0	(24.4) ^4^	70.1	(23.9)	190.2	(33.7) ^1,2,3,4^
(n)										
PL	66.9	(11.0) ^2,4^	66.4	(18.1) ^4^	69.0	(14.8) ^1,2,4^	46.7	(15.2)	142.1	(15.6) ^1,2,3,4^
(au)										
TDmin	85.1	(9.2) ^2,4^	72.7	(10.5) ^4^	85.4	(11.3) ^1,2,4^	65.1	(13.2)	90.8	(5.3) ^1,2,3,4^
(m/min^1^)										

Note: VEL is velocity day, INT is intensity or strength day, EXT is extensity or endurance day, PREOM is previous day to match day, OM is official match day. TD is total distance expressed in meters (m), HSR is high-intensity running distance (m), Sprint is sprint running distance (m), ACC2 is the number of accelerations at >2 m/s^2^ (n), DEC2 is the number of decelerations at <−2 m/s^2^ (n), PL (au) is player load, and TDmin is distance per min (m/min^1^). Significant differences (*p* < 0.05) are represented: 1 is higher than VEL, 2 is higher than INT, 3 is higher than EXT, and 4 is higher than PREOM.

**Table 2:** Effect size of external load variables in different training and competition sessions.

Comparison of Type of Sessions	Variables						
	TD	HSR	Sprint	ACC2	DEC2	PL	TDmin
VEL vs. OM	8.4 (ELI)	1.1 (MI)	0.7 (MI)	2.2 (VLI)	2.3 (VLI)	5.6 (ELI)	0.8 (MI)
VEL vs. PREOM	−1.8 (LD)	−1.7 (LD)	−0.8 (MD)	−1.8 (LD)	−1.8 (LD)	−1.5 (LD)	−1.8 (LD)
VEL vs. INT	−0.2 (SD)	−1.7 (LD)	−1.2 (LD)	0.9 (MI)	0.6 (MI)	0.0 (T)	−1.2 (LD)
VEL vs. EXT	0.4 (SI)	−0.7 (MD)	−0.2 (SD)	−0.6 (MD)	−0.7 (MD)	0.2 (SI)	0.1 (T)
EXT vs. OM	6.6 (ELI)	2.1 (VLI)	0.9 (MI)	2.7 (VLI)	3.1 (VLI)	4.8 (ELI)	0.8 (MI)
EXT vs. PREOM	−1.9 (LD)	−1.0 (MD)	−0.7 (MD)	−1.2 (LD)	−1.2 (LD)	−1.5 (LD)	−1.7 (LD)
EXT vs. INT	−0.5 (MD)	−1.1 (MD)	−1.1 (MD)	1.4 (LI)	1.2 (LI)	−0.2 (SD)	−1.2 (LD)
INT vs. OM	6.2 (ELI)	3.7 (VLI)	2.4 (VLI)	1.0 (MI)	1.4 (LI)	4.5 (ELI)	2.3 (VLI)
INT vs. PREOM	−1.2 (LD)	0.4 (SI)	0.6 (MI)	−2.3 (VLD)	−2.2 (VLD)	−1.2 (LD)	−0.6 (MD)
PREOM vs. OM	8.7 (ELI)	4.0 (ELI)	1.8 (LI)	3.7 (VLI)	4.2 (ELI)	6.2 (ELI)	2.8 (VLI)

Note: ELD is extremely large decrease, VLD is very large decrease, LD is large decrease, MD is moderate decrease, SD is small decrease, T is trivial, SI is small increase, MI is moderate increase, LI is large increase, VLI is very large increase, ELI is extremely large increase. VEL is velocity day, INT is intensity or strength day, EXT is extensity or endurance day, PREOM is previous day to match day, OM is official match day. TD is total distance expressed in meters (m), HSR is high-intensity running distance (m), Sprint is sprint running distance (m), ACC2 is the number of accelerations at >2 m/s^2^ (n), DEC2 is the number of decelerations at <−2 m/s^2^ (n), PL (au) is player load, and TDmin is distance per min (m/min^1^).

## Data Availability

The datasets generated by and/or analyzed during the current study are not publicly available due to ethics and privacy requirements, but they are available from the corresponding author upon reasonable request.
